# Effect of Patient Clinical Variables in Osteoporosis Classification Using Hip X-rays in Deep Learning Analysis

**DOI:** 10.3390/medicina57080846

**Published:** 2021-08-20

**Authors:** Norio Yamamoto, Shintaro Sukegawa, Kazutaka Yamashita, Masaki Manabe, Keisuke Nakano, Kiyofumi Takabatake, Hotaka Kawai, Toshifumi Ozaki, Keisuke Kawasaki, Hitoshi Nagatsuka, Yoshihiko Furuki, Takashi Yorifuji

**Affiliations:** 1 Department of Epidemiology, Graduate School of Medicine, Dentistry and Pharmaceutical Sciences, Okayama University, Okayama 700-8558, Japan; lovescaffe@yahoo.co.jp (N.Y.); yorichan@md.okayama-u.ac.jp (T.Y.); 2 Department of Orthopedic Surgery, Kagawa Prefectural Central Hospital, Kagawa 760-8557, Japan; me424108@s.okayama-u.ac.jp (K.Y.); kkeisuke@a1.mbn.or.jp (K.K.); 3 Systematic Review Workshop Peer Support Group (SRWS-PSG), Osaka 530-000, Japan; 4 Department of Oral and Maxillofacial Surgery, Kagawa Prefectural Central Hospital, Kagawa 760-8557, Japan; furukiy@ma.pikara.ne.jp; 5 Department of Oral Pathology and Medicine, Graduate School of Medicine, Dentistry and Pharmaceutical Sciences, Okayama University, Okayama 700-8558, Japan; pir19btp@okayama-u.ac.jp (K.N.); gmd422094@s.okayama-u.ac.jp (K.T.); de18018@s.okayama-u.ac.jp (H.K.); jin@md.okayama-u.ac.jp (H.N.); 6 Department of Radiation Technology, Kagawa Prefectural Central Hospital, Kagawa 760-8557, Japan; he421738@s.okayama-u.ac.jp; 7 Department of Orthopaedic Surgery, Graduate School of Medicine, Dentistry and Pharmaceutical Sciences, Okayama University, Okayama 700-8558, Japan; tozaki@md.okayama-u.ac.jp

**Keywords:** patient variables, osteoporosis, deep learning, convolutional neural network, ensemble model, effect size

## Abstract

*Background and Objectives*: A few deep learning studies have reported that combining image features with patient variables enhanced identification accuracy compared with image-only models. However, previous studies have not statistically reported the additional effect of patient variables on the image-only models. This study aimed to statistically evaluate the osteoporosis identification ability of deep learning by combining hip radiographs with patient variables. *Materials andMethods*: We collected a dataset containing 1699 images from patients who underwent skeletal-bone-mineral density measurements and hip radiography at a general hospital from 2014 to 2021. Osteoporosis was assessed from hip radiographs using convolutional neural network (CNN) models (ResNet18, 34, 50, 101, and 152). We also investigated ensemble models with patient clinical variables added to each CNN. Accuracy, precision, recall, specificity, F1 score, and area under the curve (AUC) were calculated as performance metrics. Furthermore, we statistically compared the accuracy of the image-only model with that of an ensemble model that included images plus patient factors, including effect size for each performance metric. *Results*: All metrics were improved in the ResNet34 ensemble model compared with the image-only model. The AUC score in the ensemble model was significantly improved compared with the image-only model (difference 0.004; 95% CI 0.002–0.0007; *p* = 0.0004, effect size: 0.871). *Conclusions*: This study revealed the additional effect of patient variables in identification of osteoporosis using deep CNNs with hip radiographs. Our results provided evidence that the patient variables had additive synergistic effects on the image in osteoporosis identification.

## 1. Introduction

Osteoporosis is a socially important disease with a high incidence in the aging society and is one of the risk factors for fragility fractures [1,2]. The global standard test for diagnosing osteoporosis is estimating bone mineral density (BMD) at the proximal femur and lumbar spine using dual-energy X-ray absorptiometry (DXA). The disadvantages of DXA include potential measurement errors and uncertainty caused by the nearby soft tissues [3], radiation exposure, and high medical costs [4].

Attempts to diagnose osteoporosis via different approaches with other modalities, such as bone morphology and bone parameters based on X-rays have been reported [5,6]. Recent review articles have reported that artificial intelligence (AI) technology developments have led to efficient applications in osteoporosis identification [7,8]. A few studies have reported osteoporosis identification analysis from hip radiographs with machine learning or deep learning (DL) [9,10,11]. Yamamoto et al. reported that convolutional neural network (CNN) models diagnosed osteoporosis for hip radiographs with high accuracy, and the diagnostic ability improved further with the addition of clinical patient variables [11].

In clinical settings, clinicians consider patient factors, examine the images, assume differential diagnoses, and reach a definitive identification. In all decision processes, clinicians use patient factors when estimating and enhancing the pre-test probability. Similarly, diagnostic studies using DL have reported that diagnostic accuracy is higher when the patient variables and images are combined [12]. However, most studies reported improved results when some difference was attained by simple subtraction of the diagnostic accuracies [13,14,15,16]. Moreover, few studies have compared the statistical methods [17]. To our knowledge, previous studies have not statistically reported the additional effect of patient variables on the image-only models in osteoporosis identification using AI.

We aimed to compare the diagnostic ability of osteoporosis using DL with hip radiographs alone and in combination with patient variables. We hypothesized that combining image features with patient variables would enhance the diagnostic ability of osteoporosis with a statistical difference. Such significant difference would clarify the importance of adding patient variables and contribute to the future development of AI diagnostic research in osteoporosis.

## 2. Materials and Methods

### 2.1. Study Design

This study was a single-center retrospective study of DL identification accuracy. The aim of our study was to identify osteoporosis from a dataset segmented from hip radiographs using several residual neural networks (ResNets), types of CNNs. Supervised learning was selected as the DL method. We compared the identification accuracy of DL from hip radiographs only and DL of ensemble models in which clinical variables extracted from clinical records were added to the data set.

### 2.2. Data Collection

Clinical and imaging data from March 2014 to February 2021 were used retroactively. The subjects of this study were 1699 consecutive patients aged 60 years or older who took hip radiographs and received DXA at our hospital 6 months before and after the date of hip radiography.

We excluded the following images: osteoarthritis with femoral head deformity (*n* = 134), unclear or poor images (*n* = 82), images showing artificial objects made of materials such as metal (*n* = 58), calcifications (*n* = 40), femoral bone deformities following prior fractures (*n* = 29), external rotations (*n* = 4), and pathological fractures (*n* = 1). Thus, 1699 hip radiographs were retained for further DL analysis.

### 2.3. Data Preprocessing

**Figure 1:**
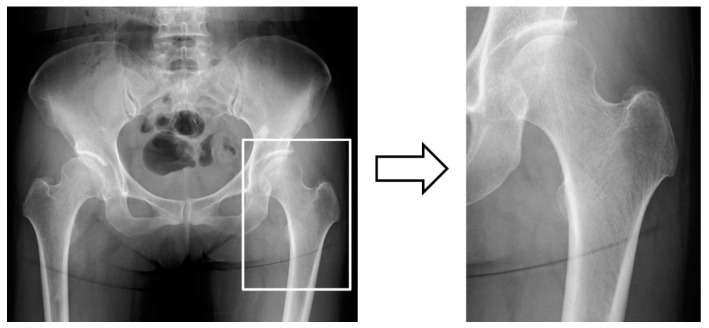
Crop method as data preprocessing. The manually cropped area perfectly imitated the osteoporosis identification range obtained using the dual-energy X-ray absorptiometry (DXA) method.

Simple hip radiographs of each patient were used to acquire the digital images. All digital images were output in tagged image file format (TIFF) format (size: 2836 × 2373, 2836 × 2336, and 2832 × 2836 pixels) from our hospital’s picture archiving and communication system (HOPE Dr ABLE-GX, FUJITSU Co., Tokyo, Japan). From the images, we segmented the hip joint area. Each orthopedic surgeon among six orthopedic surgeons processed one image under the supervision of an orthopedic expert. Six orthopedic surgeons manually cropped areas of interest in hip radiographic images using Photoshop Elements (Adobe Systems, Inc., San Jose, CA, USA). The appropriate cropped range has been selected for each hip image. The side of the hip measured using DXA was selected as the cropped side in the pre-analysis image-cropping method. The method of cropping the images was the same as that used in our previous study [11]. As with the DXA measurement, the line of the femoral head and the lower edge of the lesser trochanter were selected and cropped. The cropped areas completely imitated the osteoporosis identification range obtained using the DXA method (Figure 1). Cropped images were saved in portable network graphics (PNG) format. All orthopedic surgeons who performed the trimming were unaware of the patient’s BMD status.

### 2.4. Identification of Osteoporosis

In this study, osteoporosis was diagnosed from the hip joint using the DXA method. The parameters investigated included the automatically generated BMD (g/cm^3^) and the T-score, which were performed at the hip using DXA (HOLOGIC Horizon-A, Apex software version 13.6.0.4, Bedford, MA, USA) by trained personnel using equal measurement routines. Standard position measurements were adopted and the scanned images complied with the following criteria [18]: The hip joint is located in the center of the image, with an internal rotation of 15° to 25°, with the femoral neck, head, and greater trochanter completely within the image. The measurement was normally performed at the left hip; when the left hip had a high degree of deformity or a metal implant, the right hip was selected.

The parameters investigated included the automatically generated BMD (g/cm^2^) and T-score. Osteoporosis was diagnosed when the T-score of BMD obtained by DXA was −2.5 or lower, according to the World Health Organization diagnostic criteria [19].

### 2.5. Clinical Variables

Patients in the high-risk group of osteoporosis are generally female, older, and have a lower body mass index (BMI) [20]. Although there are many other patient variables, age, gender, and BMI were selected in this study as easily identifiable patient factors. BMI was calculated by dividing the weight in kilograms by the square of the height in meters (kg/m^2^). Weight and height were recorded at the same time as the BMD measurement. Table 1 shows the demographic characteristics of the patients included in this study.

### 2.6. CNN Architecture

In this study, the DL analysis was performed using the standard CNN model ResNet [21], which was proposed by He et al. The residual learning mechanism that is characteristic of ResNet is a common, easy-to-optimize, and effective training method for deep CNN architectures. In addition, it is a mechanism that solves the decrease in accuracy due to deepening of the layer, and a typical ResNet contains 18, 34, 50, 101, or 152 layers.

For model construction, it is effective to use the weight of the existing model as the initial value of additional learning and fine-tuning [22]. Therefore, all ResNet CNNs were trained using transfer learning with fine-tuning employing pre-trained weights from ImageNet database [23]. DL analysis was implemented using a PyTorch DL framework and Python language.

### 2.7. Architecture of the Ensemble Model

**Figure 2:**
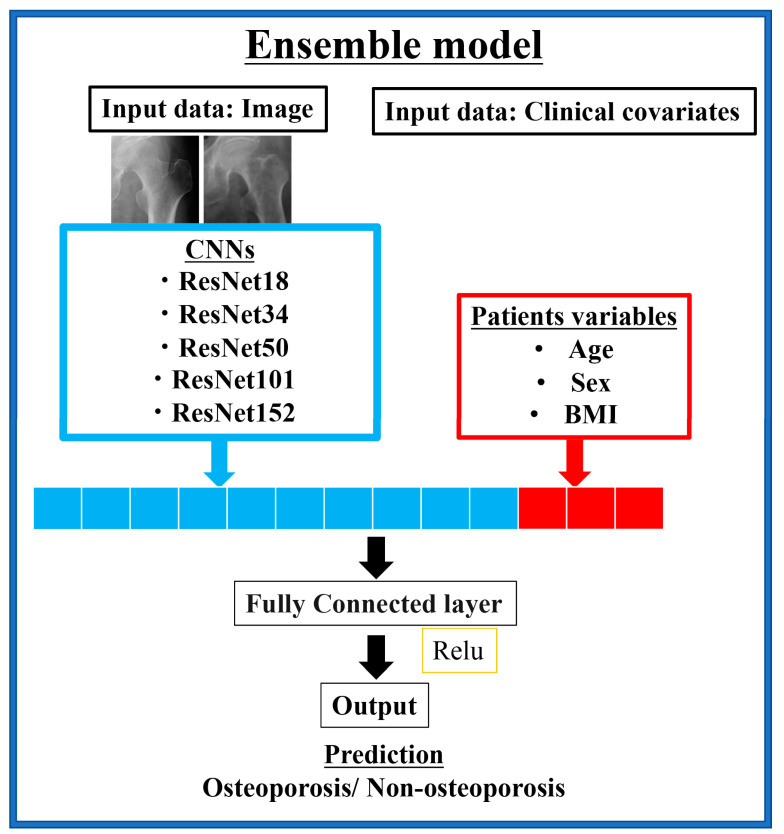
Deep neural network architecture that combines image data and clinical patient variables.

In addition to DL analysis using hip joint image data only, we constructed an ensemble model that added the clinical variables of the patient. In preparation for DL analysis, we preprocessed the patient’s structural data. Age and BMI were converted to mean normalization, and gender was converted to a one-hot vector representation. As a result, a 1 × 4-dimensional vector was created. The 1D reformed results extracted from the CNN convolution layer of the image were combined with the 1 × 4 D data created from the structural data. The image data processed by the CNN and the combined data with clinical variables were then passed as a fully connected layer. The prediction of the final osteoporosis identification model was output using the rectified linear unit activation function (Figure 2).

### 2.8. Data Augmentation

In this study, various types of data augmentation techniques were adopted to prevent overfitting. When using training data during image training, the data extension was applied only to the training image data when the images were retrieved in batches. The training image was randomly rotated in the range of −25 degrees to +25 degrees and flipped with a 50% vertical and 50% horizontal probability. Darkness and contrast were randomly changed from −5 to +5%. Each training image was processed with a 50% chance of data augmentation.

### 2.9. Dataset

The CNN model training was performed using k-fold cross-validation in the model training algorithm. The images selected as the dataset were split using a stratified k-fold that split the training, validation, and test data while maintaining the correct label percentages. The training algorithm used k = 4 for k-fold cross-validation to avoid overfitting and bias and to minimize the generalization error. The test data consisted of 425 images. In each fold, the dataset was randomly divided into separate training and validation sets at a ratio of 8:1. The validation dataset selected was independent of the training fold and was used to assess the training status. After completing this one model training step, similar validations were performed four times, each with different test data.

### 2.10. Identification Process of the DL System

Each ResNet model was trained and analyzed using a 64-bit Ubuntu 16.04.5 LTS operating system with 8GB memory and NVIDIA GeForce GTX 1080(Nvidia Co., Santa Clara, CA, USA), 8GB graphics processing unit. In hyperparameter of this study, the optimizer used stochastic gradient descent. Learning rates of 0.001 and momentum of 0.9 were used. All images were resized to 128 × 128 pixels. All models analyzed a maximum of 100 epochs. Early stopping methods were adopted to prevent overfitting. This early stop method decides to stop learning if the validation error is not updated 15 times in a row.

### 2.11. Performance Metrics

The accuracy, precision, recall, specificity, and F1 score of the test dataset were calculated using the confusion matrix as a performance metric. In addition, the area under the curve (AUC) was measured from the receiver operating characteristic curve. This is related to the function of the classifier to avoid misidentification.

### 2.12. Statistical Analysis

The differences between image-only and ensemble model performance metrics were evaluated in JMP Statistics Software Package Version 14.2.0 for Macintosh (SAS Institute Inc., Cary, NC, USA). The significance level was set to *p* < 0.05. Parametric tests were performed based on the results of the Shapiro-Wilk test. The difference between the CNN model using images only and ensemble model with patient variables added was calculated for each performance metric using the *t*-test; effect sizes were calculated for the non-parametric tests and were classified as follows: 0.2 was a small effect, 0.5 a medium effect, and 0.8 a large effect [24].

## 3. Results

### 3.1. Prediction Performance

#### 3.1.1. Performance of Hip Radiographic Image-Only Models

Table 2 shows the performance metrics of each ResNet model using only hip radiographic images. ResNet-152 scored the highest in accuracy, AUC score, precision, and F1 score. Recall and specificity were the highest for ResNet 101 and ResNet 50, respectively.

#### 3.1.2. Performance of Ensemble Models

The highest accuracy and AUC score were achieved by ResNet50, precision and specificity by ResNet34, recall by ResNet152, and F1 score by ResNet101 (Table 3).

### 3.2. Comparison of the Image-Only and Ensemble Models

Table 4 shows the evaluation of the differences between the radiographic image-only and ensemble models in the respective performance metrics. The calculation method is ensemble models minus the radiographic image-only models. The AUC improved for all ResNets. ResNet34 improved in all performance metrics, and the addition of patient variables improved accuracy.

In addition, we compared two groups of radiographic image-only models and ensemble models of each performance metric in ResNet34. Table 5 shows the results of 4-fold cross-validation evaluation performed 30 times. In AUC, the ensemble model was significantly improved over the image-only model. Regarding the effect size, the AUC was 0.871, which was an effect size that could be classified as a large effect.

## 4. Discussion

This DL study demonstrated that adding routinely available patient variables to image-only models improved their diagnostic accuracy of osteoporosis. The mean AUC score was significantly improved (difference: 0.004; 95% CI: 0.002 to 0.0007; *p* = 0.0004). The patient variables had additive synergistic effects on the image in osteoporosis identification in this DL study.

These results are consistent with those of previous studies in other fields [13,15,16,25,26]. However, performance metrics other than AUC in this study were not improved in some CNN models. The results were similar to those of a diagnostic study of diabetic retinopathy using machine learning [16]. We speculate that the diagnostic accuracy improved due to the amount of essential information and the quality of patient variables that cannot be extracted and interpreted from images alone.

The AUC scores significantly improved. The results with a relatively high AUC score suggest that the image model with patient variables offers a high discriminative power for diagnostic tests [27]. A few previous AI studies reported that the additional patient variables on the image improved the AUC by 2%–4% [14,16]. It is evident that diagnostic accuracy should be as high as possible in a diagnostic test analysis, but it is unclear how much clinical benefit would be provided by such statistical advantage.

In this study, we measured the effect size of an ensemble model of patient variables. Effect size is an indicator of the effectiveness of experimental results and the strength of relationships between variables. In this study, the effect size in AUC for osteoporosis identification was 0.871, which was classified as a large effect. Since few reports have calculated the sizes of such effects based on comparisons between DL models [28], we are confident that our study will play a role as a basic research that helps determine sample sizes for studies in the future.

The strength of this study over previous studies is that the additional effect of the patient factor was statistically assessed in a clinical risk patient population. The applicable patient group in this study was as close as possible to a real-world setting. To our knowledge, this is the first study to statistically clarify the additional effects of patient variables in osteoporosis identification using DL. In addition, the calculated effect size can be used to estimate the sample sizes for future studies. It is suitable to evaluate results statistically rather than simply by comparing different values in the academic research field.

This study has some limitations. First, the selection of patient factors was not assessed. We selected three patient factors in this study based on a previous study [11]. In selecting and deciding on the patient factors, we believed it was important to select a few simple and easy-to-collect factors if we were to prepare for real-world application. A machine learning study reported some osteoporosis risk prediction variables, such as the duration of menopause and diabetes mellitus [29]. In future studies, the selection of patient factors that predominantly influence osteoporosis identification needs to be thoroughly examined. Second, we analyzed the diagnostic accuracy of a limited selection of CNN models. CNN models are being developed at a very fast pace. We must select an appropriate model for handling high-quality images and patient variables. This will need to be validated using various CNN models. Third, we tested only the ResNet34 model with 30 cycles and found a statistically significant difference. Deeper networks require more parameters and take more time; therefore, we were not able to use them in this study. Further research should examine more CNN models and compare the confidence intervals of the differences. We speculate that appropriate models will be identified for clinically required diagnostic accuracy in each situation. Fourth, in this study, we used Photoshop to manually crop, but there are slight differences between workers within the range of the crop. In order to develop a better osteoporosis detection model, it is necessary to further study the range of crop, the difference in resizing, and the processing of padding. As a final goal, it will be necessary to develop and study a method for automatically cutting out from the hip radiographs. Fifth, we could not consider sample size in the method because previous studies did not report effect size or clinical importance difference. In this study, we reported each effect size on each performance metric. Therefore, researchers in the further research can conduct sample size calculation. Finally, we did not evaluate the external validity of our models. In different facilities and settings, the method and quality of radiographs are different. Residual overfitting of our single institutional data might not be applied to other institutional datasets, although we adopted some meticulous methods to prevent overfitting. In addition, people of different races and from different regions have different bone morphologies, and the degree of influence of patient factors will be different [30]. Big data from multicenter studies will enhance external validity and aid further research.

## 5. Conclusions

We have revealed the additional effects of patient variables in diagnosing osteoporosis using deep CNNs with hip radiographs. In particular, we found a statistically significant improvement in AUC scores.

## Figures and Tables

**Table 1:** The clinical and demographic characteristics of the patients.

	Osteoporosis	Non-Osteoporosis	
	(T-score ≤ −2.5)	(T-score > −2.5)	*p* value
Number of patients	909	790	
Gender			
Male (%)	148 (36.7)	255 (63.3)	<0.0001
Female (%)	761 (58.7)	535 (41.3)	
Mean age (SD)	81.6 (9.2)	76.3 (10.8)	<0.0001
BMI (SD)	20.5 (3.3)	22.9 (3.6)	<0.0001

Abbreviations: BMI: body mass index, SD: standard deviation.

**Table 2:** Prediction performance of hip radiographic images only models.

	Accuracy	95%CI	AUC	95%CI	Precision	95%CI	Recall	95%CI	Specificity	95%CI	F1	95%CI
ResNet18	0.792	0.768–0.817	0.883	0.860–0.907	0.842	0.763–0.921	0.761	0.617–0.905	0.828	0.715–0.941	0.795	0.740–0.849
ResNet34	0.793	0.772–0.814	0.886	0.865–0.912	0.829	0.782–0.877	0.777	0.666–0.887	0.813	0.726–0.899	0.800	0.761–0.838
ResNet50	0.798	0.753–0.842	0.885	0.866–0.905	0.841	0.789–0.893	0.768	0.678–0.857	0.832	0.762–0.901	0.802	0.752–0.851
ResNet101	0.816	0.786–0.846	0.896	0.860–0.933	0.833	0.770–0.897	0.823	0.767–0.879	0.807	0.710–0.905	0.827	0.804–0.850
ResNet152	0.822	0.778–0.865	0.900	0.869–0.931	0.844	0.811–0.876	0.818	0.742–0.895	0.825	0.784–0.866	0.830	0.783–0.877

Abbreviations: CI: confidence interval, AUC: area under the curve.

**Table 3:** Prediction performance of models with hip radiographic images and clinical patient variables.

	Accuracy	95%CI	AUC	95%CI	Precision	95%CI	Recall	95%CI	Specificity	95%CI	F1	95%CI
ResNet18	0.788	0.772–0.804	0.885	0.872–0.899	0.828	0.752–0.903	0.77	0.658–0.882	0.809	0.676–0.941	0.795	0.767–0.822
ResNet34	0.809	0.779–0.838	0.897	0.876–0.919	0.851	0.7802–0.899	0.783	0.660–0.907	0.838	0.755–0.921	0.813	0.766–0.860
ResNet50	0.812	0.785–0.840	0.906	0.881–0.931	0.823	0.790–0.857	0.828	0.728–0.928	0.794	0.723–0.864	0.824	0.786–0.863
ResNet101	0.809	0.803–0.814	0.897	0.879–0.916	0.847	0.809–0.886	0.785	0.727–0.844	0.835	0.776–0.894	0.894	0.799–0.830
ResNet152	0.806	0.781–0.831	0.9	0.880–0.920	0.815	0.742–0.889	0.833	0.708–0.958	0.776	0.639–0.913	0.821	0.786–0.855

Abbreviations: CI: confidence interval, AUC: area under the curve.

**Table 4:** Differences in prediction performance due to the addition of clinical patient variables.

	Accuracy	AUC	Precision	Recall	Specificity	F1
ResNet18	−0.004	0.002	−0.014	0.009	−0.019	0.000
ResNet34	0.016	0.009	0.022	0.006	0.025	0.013
ResNet50	0.014	0.021	−0.018	0.060	−0.038	0.022
ResNet101	−0.007	0.001	0.014	−0.038	0.028	0.067
ResNet152	−0.016	0.000	−0.029	0.015	−0.049	−0.009

Abbreviations: AUC: area under the curve. The difference was obtained by subtracting the performance of image-only model from the model using clinical patient variables.

**Table 5:** Image-only model and ensemble model of each performance metric in ResNet34.

	Image Only	95%CI	Ensemble Model	95%CI	Upper Confidence Limit	Lower Confidence Limit	*p* Value	Effect Size
Accuracy	0.797	0.795–0.800	0.800	0.798–0.803	0.000	−0.007	0.061	0.483
AUC	0.887	0.885–0.889	0.892	0.890–0.894	−0.002	−0.007	0.0004	0.871
Precision	0.820	0.816–0.825	0.821	0.816–0.826	0.007	−0.007	0.894	0.035
Recall	0.799	0.792–0.807	0.806	0.799–0.814	0.004	−0.018	0.217	0.320
Specificity	0.794	0.786–0.803	0.793	0.785–0.802	0.013	−0.011	0.848	0.050
F1	0.807	0.805–0.810	0.811	0.809–0.814	0.000	−0.008	0.059	0.487

Abbreviations: CI: confidence interval, AUC: area under the curve.

## Data Availability

Not applicable.
